# Improving Nutrition and Nutrition Education in the Burn Unit of a Developing Country: A Qualitative Study

**DOI:** 10.3390/ebj6010015

**Published:** 2025-03-10

**Authors:** Jonathan Bayuo, Joyce Pwavra, Jephtah Davids, Anita Eseenam Agbeko, Paa Ekow Hoyte-Williams, Frank Bediako Agyei, Pius Agbenorku

**Affiliations:** 1 School of Nursing and Midwifery, Presbyterian University, Agogo 23321, Ghana; frankagyei89@gmail.com; 2 School of Nursing and Midwifery, University of Ghana, Legon 23321, Ghana; paladaga1@gmail.com; 3 Department of Nutrition, University of Ghana, Legon 23321, Ghana; jephtahdavids@gmail.com; 4 Directorate of Surgery, Komfo Anokye Teaching Hospital, Kumasi 23321, Ghana; aeagbeko@gmail.com; 5 Division of Burns, Plastic, and Reconstructive Surgery, Directorate of Surgery, Komfo Anokye Teaching Hospital, Kumasi 23321, Ghana; paa_ekow@hotmail.com; 6 School of Medicine and Dentistry, Kwame Nkrumah University of Science and Technology, Kumasi 23321, Ghana; pius.agbenorku@gmail.com

**Keywords:** burns, education, nutrition

## Abstract

Burn injuries can trigger a series of metabolic and catabolic responses that exert significant impacts on an individual’s nutritional status, necessitating continuous nutritional support and education to aid recovery. However, burn units in developing countries often face resource limitations that can negatively affect these needs. This study aimed to explore the challenges related to post-burn nutrition and nutrition education in our burn unit and identify ways to improve the situation. An interpretive description approach was used, and convenience sampling recruited fifty-three participants, including 18 adult burn survivors and their primary caregivers (each as a single dyad), 10 informal caregivers of paediatric burn survivors, and 25 burn care staff. The data were analysed through thematic analysis, revealing three main themes and seven subthemes. The findings highlight an unstructured approach to nutrition and education, along with financial constraints affecting adherence. To address these issues, strategies such as using educational materials like videos and booklets/leaflets in the local language are suggested to develop relevant interventions. In conclusion, while there are concerns about nutrition and education, there are also opportunities to improve the situation.

## 1. Introduction

Burn injuries, depending on their extent, can lead to a cascade of hypermetabolism and hypercatabolism, which can negatively impact an individual’s nutritional status and the recovery process [1]. During the hypermetabolic state, the basal metabolic rate often increases to twice their normal rate, leading to severe lean mass and weight loss and placing the burned patient at a higher risk of poor recovery, delayed wound healing, and infection [2,3]. The often prolonged nature of the post-burn recovery process may suggest a longer duration of hypermetabolism, highlighting the need to pay significant attention to the nutritional needs of affected persons [4]. Indeed, optimum nutrition remains essential for burn patients and survivors [5].

Despite the great need for adequate and proper nutrition in the burn management process, developing countries are often faced with limited resources, which can impact the ability to procure nutritional supplements [6]. Indeed, several nutritional issues have been reported in various developing countries, including protein deficiency, malnutrition, nutritional anaemia, and vitamin deficiencies [7]. One study that examined the adequacy of the dietary intake of antioxidant micronutrients in a Ghanaian tertiary healthcare facility observed that the majority of the study participants did not meet the micronutrient dietary requirements, with deficiencies in vitamins A, C, and E, zinc, copper, and selenium [8]. The presence of these issues in the pre-burn state indicates the concurrent existence of burns and nutritional issues, further straining the body and creating a significant need for optimum nutrition in the post-burn period [1]. These necessitate the consideration of innovative strategies to improve nutrition, particularly using locally available items so as not to strain the finances of burn patients and their families.

Among the various strategies put forward to improve the nutritional status of burn patients, nutritional education remains paramount [1,9]. In fact, several studies have reported significant improvements in eating habits and anthropometric indices and the prevention of malnutrition among diverse patient population groups [10,11,12]. Despite these findings, a recent scoping review examining nutrition education programmes for burn survivors reported a general lack of studies reporting on burn-specific nutrition education programmes [1]. The authors therefore highlighted the need for more work in developing, implementing, and evaluating such programmes. These concerns resonate with our burn unit, where nutrition education has received limited attention. As a starting point, this study sought to engage with burn care practitioners, burn survivors, and their families to uncover existing challenges to post-burn nutrition and nutrition education and identify a way forward to improve the situation.

## 2. Materials and Methods

### 2.1. Study Design

Given the need to understand the current challenges impacting nutrition and nutritional education in our burn unit and to work towards improving the situation, we employed interpretive description. Interpretive description is a non-categorical qualitative research methodology that focuses on generating knowledge that can contribute to practice [13]. Its interpretive and inductive nature makes it particularly suitable for the current study as it can facilitate the development of a context-specific nutrition education programme. Moreover, the aspect of capturing shared realities while paying attention to nuances makes it appropriate for a study that includes both burn survivors and burn care practitioners. This study is reported according to the Consolidated Criteria for Reporting Qualitative Research (COREQ) checklist [14].

### 2.2. Study Setting

This study was undertaken in the middle belt of the Republic of Ghana, West Africa. The middle belt of Ghana covers the Ashanti, Eastern, Bono, Bono East, and Ahafo regions. Participants were recruited from a large teaching hospital that served people from the middle and northern zones of Ghana. This teaching hospital serves as the main referral healthcare facility for all forms of burn injuries across these zones. The hospital has a 1000-bed capacity with a 6-bed burn ICU and a 6-bed burns ward. The yearly admission rates range from 481 to 650 patients, with paediatric burn patients representing more than half of all cases [15]. In our setting, nutrition education is not routinely undertaken unless a patient is reported to have a poor appetite. At discharge, nurses often provide brief information on the need to maintain adequate nutrition as part of discharge teaching. In terms of nutritional support, routine nutritional assessment is not carried out following admission to the burn unit unless a surgeon requests a formal nutritional consult. Thus, nutritionists/dieticians are not readily available in our unit unless their services are requested. The hospital routinely serves three regular meals daily. Vitamin and mineral supplementation are not provided by the hospital, and, if requested, the patients need to purchase them out of pocket.

### 2.3. Participants, Sampling, and Sample Size

The participants for this study were burn survivors (second- to third-degree burns with TBSA ≥ 10%), previously admitted to the burn ICU and with a discharge status of ≥2 months, and their primary caregivers (as a dyad), who were willing to participate and reachable via phone or available for either a face-to-face or virtual meeting. We also included burn care practitioners with at least 6 months of working experience in the burn unit. In this study, a dyad is considered a single unit in qualitative terms to indicate the holistic nature of the experience rather than the fragmentary experience of two participants. Thus, a dyad of a burn survivor and a family caregiver is considered as one participant instead of two distinct participants. Burn survivors with confirmed underlying chronic illnesses and burn care practitioners who were on leave during the study period were excluded. Due to the nature of the study and the challenges associated with reaching out to potential participants, we employed a convenience sampling approach to recruit both burn survivors and burn care practitioners. Although we were guided by the principle of data saturation, a sample size of up to 24 was considered adequate [16].

### 2.4. Data Collection

Semi-structured interviews were held either face to face or virtually with participants using an interview guide formulated by the research team. The interview guide was piloted among two burn survivors by the lead author, following which minor changes were made. The interviews were undertaken by research team members (JB and JD) who had extensive training in qualitative methods. Following recruitment, the lead author phoned the participant to arrange a mutually agreeable date, venue, and time for the interview. On the day of the interview, the intention to participate was confirmed and consent obtained. All interviews were undertaken in the presence of only one research team member. All participants were encouraged to talk freely about their experiences. The interviews were undertaken either in the Akan or English language, based on the preference of the participant. Emerging responses were probed further to obtain in-depth data regarding post-burn nutrition and nutrition education. All interviews were audio-recorded. Interviews continued till data saturation was attained. Follow-up interviews were also undertaken to clarify emerging issues.

### 2.5. Data Analysis

All interview recordings in English were transcribed verbatim using the Trint automated transcription software version 1. For the interviews in the local dialect, a native speaker who was also fluent in English translated them verbatim. All interviews and follow-up interviews were collated as one complete transcript. Following review, we employed thematic analysis to analyse the data. The thematic analysis approach sought to identify, interpret, and report patterns in the data through the six steps of becoming familiar with the data, generating initial codes, searching for themes, reviewing themes, defining themes, and writing up [17]. Each manuscript was read and re-read to ensure familiarity with its content, following which we inductively undertook line-by-line coding to formulate an initial coding frame. The coding frame was reviewed and applied to two randomly selected interview transcripts, following which the remaining transcripts were coded. Following the formulation of codes, similar codes were subsequently grouped as subthemes. Emerging subthemes were reviewed and grouped to develop higher-order themes.

### 2.6. Trustworthiness and Methodological Rigour

Credibility, confirmability, transferability, and dependability underpinned the approach to ensuring trustworthiness in this study [18]. The strategies included the use of probes and prompts, undertaking follow-up interviews, and member checking.

### 2.7. Ethical Considerations

Ethical approval for this study was granted by the Institutional Review Board of the Komfo Anokye Teaching Hospital (KATHIRB/AP/014/21). All guidelines regarding the ethical conduct of research were followed.

## 3. Results

Fifty-three participants participated in this study, consisting of 18 adult burn survivors and their primary caregivers (as a dyad), 10 informal caregivers of paediatric burn survivors (all mothers), and 25 burn care staff. The majority of the burn survivors were male (n = 12) and the majority of the burn care staff were female (n = 18). All participants had survived burns ≥10% TBSA. The age of the participants ranged from 22 to 62 years. The majority of the burn survivor participants (n = 12) had survived thermal burns with no inhalational injuries; scalds (n = 5); and electrical burns (n = 1). The working durations of the burn care staff participants ranged from 8 months to 8 years. The duration of the first round of interviews was between 18 and 32 min, and the follow-up phone interviews lasted between 4 and 9 min. From the data, three themes and seven subthemes emerged, demonstrating the shared realities across the study (Table 1).

### 3.1. Theme 1: Existing Nutrition and Nutrition Education Support for Burn Patients

This theme encompasses the available nutritional and educational support for burn patients/survivors and their families. The subthemes that emerged were (1) nutrition and nutrition education in “generic” terms and (2) the lack of a structured approach to post-burn nutrition.

#### 3.1.1. Nutrition and Nutrition Education in Generic Terms

All participants underscored the importance of nutrition in the post-burn recovery process, similarly to the critical need for adequate pain control and wound management. Regarding the food provided in the hospital, burn survivors and their families felt that it was same as the food that they had consumed throughout their lives; sometimes, what they had eaten previously was even better than the meals provided in the hospital. The meals offered by the hospital were considered unappealing, although the burn care staff emphasised that it offered some nutritional benefits for the post-burn recovery process.

*“Of course, nutrition is important. Even when there is no injury, it is important, and, after the injury, it becomes even more important so they can get well”*.

(Burn Surgeon, 004)

*“I felt it was the same food we eat at home, nothing really special about it. At home, I can cook it myself but here we don’t even know who does the cooking and serving the food to us”*.

(Adult Burn Survivor, 012)

*“The food did not really look that nice but we had to take it and feed the child. The choice was limited and we had to take it like that”*.

(Informal Caregiver, 009)

The burn care practitioners, burn survivors, and family caregivers underscored the availability of some form of nutrition education in the burn unit and following discharge, albeit in general terms; it did not entirely meet their expectations and was not tailored to the survivor’s unique circumstances. For burn survivors and their family caregivers, the education received was viewed as what they came across in everyday life, was not personalised to their individual circumstances, and did not meet their expectations. In other instances, the participants felt that the suggested nutritional recommendations were not feasible, were subjective, and required more objective information regarding why they were necessary.

*“…they only told me to give him eggs everyday but he was not eating well and I could not force the egg on him”*.

(Informal Caregiver, 010)

*“Yes, we do advise them to feed on high-protein and high-calorie diet because they are not discharged with the wounds healed, so, to aid the healing process, we tell them that, when they go, they should eat a high-protein and -calorie diet so that the protein aids the healing process, so they should be much concerned about it”*.

(Burn Nurse, 010)

*“at times we do sight some of the foods that are available to them, so when you eat, let’s say, when you eat 2 or 3 boiled eggs, let’s say, 2 to 3 times in a day, it serves as a source of protein… we inform them that these are foods that, when they eat them, they get such proteins from them. So, at times, with the foods surrounding them, we just tell them that the nutritional content that we are looking for is in the foods that surround them, so they should do much and eat”*.

(Burn Surgeon, 002)

Whereas the burn care staff felt that they were passing on information about available supplements that could help the patients, the burn survivors felt that they would have preferred an approach that was tailored to their needs and within their financial means and did not overwhelm them.

*“Well, we tell them that when the wounds are not really healed, they should continue eating well, a well-balanced diet, and increase their intake of protein and vegetables and take in a lot of fibre foods”*.

(Burn Surgeon, 003)

*“Some of the things they told me to buy and add to my food, I didn’t know where to get them. I wished they had told me more about what I need to eat and recover well”*.

(Adult Burn Survivor, 018)

#### 3.1.2. Lack of a Structured Approach to Post-Burn Nutrition

The participants underscored the general lack of teaching aids and a structured approach to post-burn nutrition education. Most often, the task of providing nutrition education fell to the nurses. Generally, the burn care staff highlighted the existence of aids for some tasks, such as pain management, but these were absent for nutrition education. This made it difficult for them to offer objective, tailor-made information to the burn survivors and their families prior to discharge.

*“Unless the nurses prompt the doctors to do it, sometimes the nurses have to do it themselves. They have to go and tell them verbally, and they will also say they need a written ‘something’ to show it is coming from the doctors; the patient really needs nutritional support”*.

(Burn Nurse, 011)

*“Actually, I think it should have been done by the nutritionist, but since they are not readily available, it is the nurses that do it, so it is not structured or detailed. We just give an overview of what we know, what will be good for the patient, looking at the patient’s age. Maybe, if it is a paediatric, we know that maybe cereals and a lot of proteins will be good for the child. For adults, too, protein but a lot of calories and carbohydrates for energy. It is just like we give a general idea of what we have compared to what the nutritionist would have given to the patient”*.

(Burn Surgeon, 005)

Following discharge, the burn care staff highlighted that limited to no information is provided to burn survivors and their families regarding nutrition. When some information was provided, it was usually scant, with a significant amount of information on wound care and the need to return for medical follow-ups.

*“Following discharge, I think we educate them on their follow-ups; we also educate them on proper wound dressing, but not much attention is placed on their nutritional needs. Personally, I don’t remember the last time I educated patients following discharge on nutrition, but I think it is something that we should also start looking at because sometimes we may see that the wound is fully healed and the patient can go home but we also need to pay attention to their nutrition”*.

(Burn Surgeon, 003)

*“I haven’t seen any staff do that; like I said, the basis for the education post-discharge is generally on wound care and sometimes coping, how they will cope with the obvious disability that they will encounter from the pain, but nutrition is not something we do”*.

(Burn Surgeon, 001)

*“Even when the patient is on the ward sometimes, we do it, but post-discharge is something that we don’t do. So, I think we can first and foremost be educated on the need for proper nutrition following discharge, and, when we have that adequate education on nutrition, then we can also intend give feedback to the relatives”*.

(Burn Nurse, 007)

### 3.2. Theme 2: Adherence to Nutritional Requirements

This theme encompasses the factors associated with adhering to the post-burn nutritional requirements that emerged from the data. The subthemes discussed are as follows: financial constraints, knowledge of nutritional requirements, and social support.

#### 3.2.1. Financial Constraints

All participants highlighted financial issues as a key factor that impacted both the availability and adherence to post-burn nutritional requirements. Whereas burn survivors and informal caregivers felt that it was not entirely necessary to be informed of all of the nutritional supplements that they could not afford, the burn care staff felt that they still had to highlight them to the survivors and their families in case they were able to afford them.

*“To be honest, some of the items they mentioned, when we checked the prices, we just could not afford them. We use what we have”*.

(Adult Burn Survivor, 005)

*“I still think we should tell them the proteins and calories because they need to heal quickly, and they need a lot of protein to serve as the building blocks. As they are doing on the ward, they should continue. Most of the time, they just cannot afford the items”*.

(Burn Nurse, 017)

*“I think that one differs from patient to patient. There are some that they are able to do, but there are some too that they are not really able to do because of their financial background. Because they also have to pay for dressing materials and things like that. And sometimes they can’t always do that and so it depends on the patient’s financial background, but I think most are able to do it. I just have one patient on admission with some financial issues, and she is the only one that is not always like the way you want it being done aside her. I think the ones I have helped manage since I was here, they have been okay”*.

(Burn Surgeon, 001)

For some burn survivors and informal caregivers, limited financial resources meant prioritising other aspects of care, such as obtaining wound dressing materials and medications rather than nutritional supplements. Inasmuch as the hospital food was not considered satisfying enough, it was sometimes considered better than nothing, particularly in instances of limited financial resources.

*“When the wound gets soaked, you just want them to change the dressing as soon as possible. If money is lacking, we try to get the dressing materials first before anything else so the nurses can change the dressing. It is hard, but what more can we do?”*.

(Informal Caregiver, 010)

#### 3.2.2. Knowledge of Nutritional Requirements

Being aware of the post-burn nutritional requirements was also observed to impact adherence to the survivor’s nutritional needs. The adult burn survivors and caregivers of paediatric burn survivors expressed interest in knowing not only what is required nutritionally but also why it is required and alternatives available in the local community.

*“For my daughter’s well-being, I was very interested in learning about all that I can get from the nurses and doctors. But it was not usually the case, and I did not want to ask so many questions too”*.

(Informal Caregiver, 001)

The burn care staff also underscored the fact that they needed more training to understand the nutritional requirements of burn patients and survivors. This emerged particularly from the nurses, who often undertook the task of providing nutritional education, regardless of how unstructured it may be.

*“Well, they should also give the nurses some chance to also study nutrition, since the nutritionists are not also forthcoming, because they are not only attending to burns units, they are attending to other units as well. So maybe some of the burns nurses should also be given the opportunity to study nutrition and then when they come in or burns patients are brought in, they can also give advice on what to take and what not to take. I think that is where they fall short. So, they should be given the opportunity to also go and learn”*.

(Burn Nurse, 012)

*“They have to support in sponsoring some of us to study nutrition as part of our courses and choice of courses, or maybe we organise workshops or collaborate with the nutrition department and organise some workshops and seminars for the nurses so that, when they are not around, we will know what to give and what not to give to the patients and the number of calories to be given to the patients that will help them to recover faster”*.

(Burn Surgeon, 004)

#### 3.2.3. Social Support

Social support from family members and friends emerged as a significant factor associated with availability of and adherence to post-burn nutritional requirements. Adult burn survivors highlighted that having someone to support them in terms of procuring food items and preparing food was helpful, since they were often unable to do these on their own. Additionally, they felt that the presence of another during feeding times helped to stimulate their appetite as they were encouraged to try and eat more.

*“When she was at the hospital, they made sure she got her protein intake, especially with the eggs. And even with the breakfast, where she was fed up with eating eggs, we were, you know, finding means to blend it with the porridge and stuff”*.

(Informal Caregiver, 006)

Further to the above, some burn survivors and informal caregivers mentioned the availability of support outside the immediate burn care environment, who occasionally offered nutritional advice as they journeyed through their recovery.

*“I know those things are good and I even have a friend who is a nutritionist that normally I do requests for these menu plans. Even before we got to the hospital, when he got to know that we were there, he called, he told me he will be calling one of his mates there to come around”*.

(Informal Caregiver, 001)

### 3.3. Theme 3: Strategies to Improve Post-Burn Nutrition and Nutrition Education

This theme encompasses suggestions put forward by the study participants regarding how to improve post-burn nutrition and nutrition education in our context. The subthemes are repackaging post-burn nutrition education and active follow-up support after discharge.

#### 3.3.1. Repackaging Post-Burn Nutrition Education

The burn care staff noted a great need for a nutritional management protocols that were similar to other forms of protocols, such as wound care, available in the unit. It was mentioned that the protocol should offer a structured approach to nutritional management and education, noting what the patient needs and not only emphasising protein. Moreover, the need for on-the-job nutritional training was highlighted to enable the staff to offer support even in the absence of a nutritionist or dietician.

*“Yes, there is a way, because one thing that I have realised is that, it is like we don’t, most of the nurses don’t add it to the admission process and the protocols, so the nurses on board, when we all come on board and just include it in the admission protocol, it is a must that every nurse on duty has to educate the patient on the need to eat a high-protein or high-calorie diet. I think it would go a long way to help the patient”*.

(Burn Nurse, 011)

*“Sure, I think if we have a protocol that we follow. Ghanaians basically we are meant for protocols, if we have a well-defined strategy for attending to nutrition education, I think it would help, but until now I haven’t seen such a protocol. So, if we can do it, it would help us clearly define the strategies that we can use. Because a patient needs such nutritional support”*.

(Burn Surgeon, 005)

*“We only know of the high-protein meals that the patient needs, but I believe that it is not only high-protein meals that the patient needs but it depends on the situation in which the patient finds himself, so, even inasmuch as you are providing the high-protein meals, you can also provide other sorts of meals that can help the patient to recover fully, because the whole idea is helping the patient to fully recover, so if that is something that we can do to help the patient recover fully, we can do it”*.

(Nutritionist, 001)

The burn care staff also mentioned commencing nutritional counselling as soon as the patient is admitted to the burn unit, which can continue throughout the hospitalisation period and makes it easier to transition to the post-discharge period with the knowledge gained. This was considered essential since patients could easily forget one-off discussions. Additionally, the burn care staff called for all members of the team to be involved in providing such education, rather than leaving it to the nurses. Patients and their caregivers should also be actively involved in the process.

*“As to how it should be packaged, I personally think that, on arrival, it is very important for them to have nutritional counselling, and this should be done on daily basis, because I have found that patients usually forget a lot, and it should be reemphasised to their caregivers in particular the importance of nutrition, which forms a major role in the care of the patient”*.

(Burn Surgeon, 001)

*“…so, this should be done by the doctors who will mention it, then the nurses will also come and mention it, then the nutritionist will also come and reinforce it”*.

(Burn Nurse, 002)

*“Once the patient is on admission, you don’t need to send a consult to a nutritionist to come in; they know that once the patient has been admitted with burns you need to come in to draw up a nutrition plan for the patient. So, for here, if the consult doesn’t go ahead, they don’t come”*.

(Burn Surgeon, 003)

*“I believe that it should be something which should be all-inclusive; we should include the relatives, we should include the patients, and even the hospital staff as well. Because the hospital staff must first and foremost be educated on the importance of nutrition. Because it is important when the patient is at the ward and following discharge at home also”*.

(Nutritionist, 001)

The burn survivors, informal caregivers, and burn care staff underscored the need for easy-to-read educational booklets and leaflets that could be used to deliver nutritional education. This was described as critical, particularly following discharge, when survivors and caregivers require reminders to enable them adhere to nutritional requirements. All participants highlighted a need for such a booklet to be available in commonly used local languages and English and be easily readable, with images of locally available food sources that they can readily obtain, even with limited financial resources, after discharge.

*“As for feasibility, I think it is feasible; first, it can be done while they have a burn, once they are being managed for their burn injuries, we can have education on that. If they have flyers or flyers that talk about how they should feed themselves, what they should eat and what benefits it will give to them, I think that will help right from the time they have the burn and then subsequently they can be actively pursued. I think it would be acceptable”*.

(Burn Surgeon, 004)

*“If it’s something we can easily read and understand and take home, I am ok with it”*.

(Adult Burn Survivor, 007)

*“Well, I think it would be helpful if it comes with pictures, because not all patients can read, but if it come with pictures, they will know the type of protein that will help them heal faster”*.

(Burn Nurse, 011)

*“Alright, I think we should let them know some of the food types that contain the proteins that we are talking about. If possible, we should even draw some pictures of some of the foods that we intend on giving them. Then, state what that particular food actually contains, the nutritional value that is in that particular food. And we should let them know the importance of why we are giving them the food in the leaflet”*.

(Nutritionist, 001)

*“Yes, it is very necessary, especially looking at the setup that we find ourselves in; most of them, sorry to say, are illiterate. But definitely in a household you will get someone who can read Twi but cannot read English, so, when such a person gets the Twi, let’s say a portion of the English and Twi as well, if he gets home, if the person cannot read the Twi, they can also use the English. So, we can blend the two languages. Basically, Twi and English”*.

(Burn Nurse, 013)

*“Well, if the patient can read the local language, I think we can use a local language for it. But if the patient cannot read, and it is not everybody who speaks the local language who can put it on paper, personally I find it difficult to write in my local language, so, if you ask me to write in it, I can’t write in it, though I speak it fluently”.*

The use of an educational video also emerged, which can be shown to patients or survivors and their caregivers while they are in the hospital and physiologically able to comprehend the content. Here, again, all participants suggested that the video should be created using a local language and supplemented with English, providing them with information about what they should eat, how much they should consume, and the benefits.

*“Apart from the book, also videos can be made and shared with them once they are OK to understand all that is going on. The videos can be helpful and should be entertaining as well, using the local language and English. I think it will help”*.

(Burn Surgeon, 002)

#### 3.3.2. Active Follow-Up Support

The adult burn survivors and informal caregivers recounted how they felt being left on their own following discharge, with limited to no professional support compared to when they were hospitalised. For participants who resided in other regions, they would have preferred to return to the facility for follow-up, but this was usually not possible due to financial constraints and long distances.

*“Once we left the hospital, it was like we were on our own and had to do things on our own. Sometimes, we forgot all that were taught during the hospital period. If possible, you can check on us on WhatsApp or something and remind us of what we need to be doing”*.

(Adult Burn Survivor, 005)

*“Returning to Gee [hospital] was not possible because the transport fare had to be used for her food. Better we stay home and use the money for something else”*.

(Informal Caregiver, 010)

## 4. Discussion

Nutrition remains a critical aspect in sustaining bodily functions; this is particularly important for burn survivors, who often experience significant physiological alterations due to the injury. Maintaining adequate nutrition in a resource-limited context such as ours is often associated with significant challenges. The current study employed qualitative methods to examine existing challenges and strategies to improve the situation. The study’s findings reveal significant limitations in post-burn nutrition and nutritional education, including the lack of a structured approach to post-burn nutrition and limited attention to the post-discharge period, when burn survivors and their families often felt that they were being left on their own. The findings affirm the need for a proactive approach to nutrition education with an emphasis on locally available items and active follow-up with burn survivors after discharge.

The education of patients and their significant others remains an essential part of disease management, prevention, and improved outcomes [19]. In burn care, patient education has also been highlighted as important [20]. Despite the established significance, only limited progress has been made to develop, implement, and evaluate relevant interventions [1]. The current study highlights several issues impacting nutrition education, including the lack of a structured approach, financial constraints, and a lack of awareness about how to approach nutrition education in the burn unit. These may indicate the limited attention paid to nutritional support in our setting. Burns often occur suddenly, and, in the initial phase of care, nutrition may not be a priority [21]. Thus, there is likely to be a significant emphasis on acute or critical care and wound management, with limited attention to nutritional support. In our context, nutritionists/dieticians are not routinely found in the burn unit. To use their services, the burn surgeon is required to complete a formal consultation form prior to a nutritional review. This process may take some time, leading to further delays in commencing optimum nutrition in our burn unit. Moreover, intensive nutritional supplementation is not routine practice in our unit, and any costs have to be paid out of pocket by the patient/family; several survivors would not be able to afford this. Although it has been established that a personalised approach to patient education yields better outcomes [19], the approach to nutrition education in our unit is often unstructured and expressed in very broad terms. This may offer limited utility to burn survivors and their families. Innovative strategies, such as using appropriate teaching aids, assessing the learning needs of burn survivors and their caregivers, and active follow-up, may be useful in ensuring that the education is tailored to their needs [22,23]. A potential strategy could be to use educational materials that are readily available publicly or develop a context-specific one in a language that can easily be understood by patients and their families.

In a resource-limited setting, financial constraints remain an ongoing issue, as highlighted in the current study, since patients need to cover their own expenses, such as purchasing dressing materials, paying for ICU accommodation, and purchasing medications/nutritional supplements. These can strain a patient or their family’s financial reserves. Burn care is undoubtedly expensive, with an average cost of USD 88,218 per burn patient in high-income settings [24] and USD 15,250 in a developing setting [25]. Similarly to other resource-limited settings, there is no comprehensive insurance package for burn patients admitted to the intensive care unit, requiring out-of-pocket payment. Although the exact burn care costs have not been empirically evaluated in the Ghanaian context, emerging evidence indicates that being admitted with severe burns can deplete a family’s financial resources [26,27]. These concerns draw attention to the need to employ locally available materials and examples when implementing a nutrition education program. In a similar context, the need to employ locally available materials in burns rehabilitation programmes has been highlighted in a recent cross-country study to avoid burdening patients and their families [28].

The lack of professional support following discharge also remains a significant issue worth highlighting. Similar to the current study’s findings, other studies have also reported burn survivors and their families feeling alone following discharge [4,29,30]. Undoubtedly, burns are acute; however, the protracted nature of the recovery process reflects the experience of living with a chronic disease [4]. Thus, discharge from the burn unit is not an end to treatment, warranting a smooth transition from the burn unit to the home or community [31]. Active professional follow-ups are therefore crucial to ensure that burn survivors and their families continue to receive ongoing support. In a recent telerehabilitation programme, a social media platform was used to actively reach out to burn survivors, assess their needs, implement strategies, and evaluate care at a distance [31]. This approach was considered both feasible and safe and helped to overcome concerns about long travel distances and financial issues. Such an approach can be used to implement nutrition education as a standalone intervention or as part of a rehabilitation bundle of care for burn survivors and their families. In this way, burn survivors would continue to feel supported even after discharge, and this would potentially contribute to improving outcomes.

The International Society of Burn Injuries (ISBI) Practice Guidelines for Burn Care stipulate the need to assess the nutritional status as part of the initial evaluation for all burn patients, estimating their energy requirements using standardised formulas, using a high-protein diet, and commencing oral diets or enteral feeding as soon as possible [32]. Although early oral feeding is likely to commence in our unit, this study’s findings suggest that nutritional assessment and the evaluation of energy requirements are not routinely practiced in our unit. Although the intake of eggs in a high-protein diet was observed in the current study, the lack of assessment suggests that it is unclear whether the degree of egg intake actually satisfies the high protein requirements of burn patients. These concerns resonate with the ISBI’s assertion that, in resource-limited settings, a greater emphasis is likely to be placed on supplies and equipment that has a more immediate role in patient care (such as antibiotics) [32]. Thus, there is a need to specifically allocate adequate means for the provision of nutritional support [33]. For our unit, a starting point could be ensuring the availability of a nutritionist/dietician in the burn unit around the clock, rather than having to wait for a formal consult to be completed.

A notable strength of this study is the inclusion of burn survivors, informal caregivers, and burn care providers in a single study to ascertain existing concerns regarding nutrition and nutritional education. This approach offered an opportunity to capture shared concerns and adopt an active participatory stance to improve the situation. Despite the interesting findings and notable strengths, this study focused on a burn unit in a developing country. Thus, some findings may be unique to our setting, although the in-depth discussion presented could enhance their transferability to other settings. Moreover, this study only considered participants who were available for either face-to-face or virtual meetings. This approach could have potentially excluded information-rich participants.

## 5. Conclusions

Nutrition remains a significant component of the burns management process, considering the hypermetabolic and hyperdynamic consequences of the injury, although it is faced with various challenges. Although nutrition education can help to improve nutrition, only limited progress has been made to develop such programmes. The current study uncovered existing concerns regarding post-burn nutrition and nutritional education, including the lack of a structured approach, financial constraints, and limited knowledge. Strategies to improve the situation were highlighted, which could inform context-relevant interventions to enhance nutrition and nutrition education.

## Figures and Tables

**Table 1:** Themes and subthemes.

Theme	Subthemes
Existing nutrition and nutrition education support for burn patients	Nutrition and nutrition education in generic terms Lack of a structured approach to post-burn nutrition
Adherence to nutritional requirements	Financial constraints Knowledge of nutritional requirements Social support
Strategies to improve post-burn nutrition and nutrition education	Re-packaging post-burn nutrition education Active follow-up support

## Data Availability

The original contributions presented in this study are included in the article. Further inquiries can be directed to the corresponding author(s).
